# Fire and Brimstone: The Microbially Mediated Formation of Elemental Sulfur Nodules from an Isotope and Major Element Study in the Paleo-Dead Sea

**DOI:** 10.1371/journal.pone.0075883

**Published:** 2013-10-01

**Authors:** Tom Bishop, Alexandra V. Turchyn, Orit Sivan

**Affiliations:** 1 University of Cambridge, Department of Earth Sciences, Cambridge, United Kingdom; 2 Ben Gurion University of the Negev, Beer Sheeva, Israel; Missouri University of Science and Technology, United States of America

## Abstract

We present coupled sulfur and oxygen isotope data from sulfur nodules and surrounding gypsum, as well as iron and manganese concentration data, from the Lisan Formation near the Dead Sea (Israel). The sulfur isotope composition in the nodules ranges between -9 and -11‰, 27 to 29‰ lighter than the surrounding gypsum, while the oxygen isotope composition of the gypsum is constant around 24‰. The constant sulfur isotope composition of the nodule is consistent with formation in an ‘open system’. Iron concentrations in the gypsum increase toward the nodule, while manganese concentrations decrease, suggesting a redox boundary at the nodule-gypsum interface during aqueous phase diagenesis. We propose that sulfur nodules in the Lisan Formation are generated through bacterial sulfate reduction, which terminates at elemental sulfur. We speculate that the sulfate-saturated pore fluids, coupled with the low availability of an electron donor, terminates the trithionate pathway before the final two-electron reduction, producing thionites, which then disproportionate to form abundant elemental sulfur.

## Introduction

Organic matter oxidation through a variety of electron acceptors is a key microbially mediated process in sediments [1]. The factors determining which electron acceptor is used are their relative abundance, their decrease in free energy yield when reduced and their availability [1-3]. Dissolved sulfate (SO_4_^2-^) is by far the most abundant of these electron acceptors in the marine environment, being nearly two orders of magnitude more abundant than oxygen at the sediment-water interface and subsequently responsible for over half of all organic matter remineralisation in sediments [4,5]. Bacterial sulfate reduction proceeds by the chemical reaction SO_4_^2-^ + 2CH_2_O H_2_S + 2HCO_3_^-^.

Sulfate-reducing bacteria, as a group, have broad ecological tolerances; they can endure temperatures between -1.5°C and 100°C [6] and salinities from freshwater to halite saturation [7,8]. It has been suggested through microbial-growth experiments and geochemical data (sulfur isotopes) that bacterial sulfate reduction occurs in the hypersaline (ten times seawater) Dead Sea brines, as well as in the groudwater adjacent to the Dead Sea [9,10]. Other microbial processes may also occur in this hypersaline environment, specifically methane oxidation, which may be coupled anaerobically to bacterial sulfate reduction [11]. Further isotope and major element geochemical evidence suggests that bacterial sulfate reduction also occurred in ancient hypersaline brines in the Dead Sea [12].

The primary product of bacterial sulfate reduction is typically hydrogen sulfide, which is reactive towards sedimentary iron, forming pyrite, or can be reoxidized back to sulfate or other higher valence state sulfur species [4,6]. One of the intermediate valence state sulfur species that can be the product of either sulfate reduction or sulfide reoxidation is elemental sulfur, S^0^. Elemental sulfur, also known as ‘native sulfur’, has been found in a range of natural environments, including lake sediments [13-16]. Unlike pyrite, which is largely disseminated in sedimentary rocks, elemental sulfur often forms large nodules or veins, millimeter to centimeter in size. While elemental sulfur formation is thought to be microbially-mediated, except in unique chemical environments associated with hydrothermal systems [17], what governs its formation remains enigmatic. Most of the literature suggests it is formed by partial re-oxidation of hydrogen sulfide (S^2-^) formed during bacterial sulfate reduction [14,16,18-21]. However, it remains engmatic whether sulfur nodules could also form during incomplete bacterial reduction of sulfate or as a stop point during the bacterial disproportionation of sulfur, or even during gypsum metamorphism [22-24]. One interesting observation is that sulfur nodules and veins are particularly common within gypsum sediments and rocks [14,19,22,23].

Isotopes are a powerful tool to study microbially mediated processes involving sulfur, including (but not limited to) the formation of elemental sulfur. Sulfate reducing bacteria preferentially reduce ^32^S and ^16^O bearing sulfate, partitioning heavy and light sulfur and oxygen isotopes between the reactant and the product. Sulfur isotope fractionation ranges between 2 and 70‰ with ^32^S preferentially accumulating in the product sulfide. Theoretical and experimental studies have suggested that the magnitude of sulfur isotope fractionation is a function of microbial metabolism and carbon source [25-28], rate of sulfate reduction [29], amount of sulfate available [30], and temperature [31,32].

**Figure 1:**
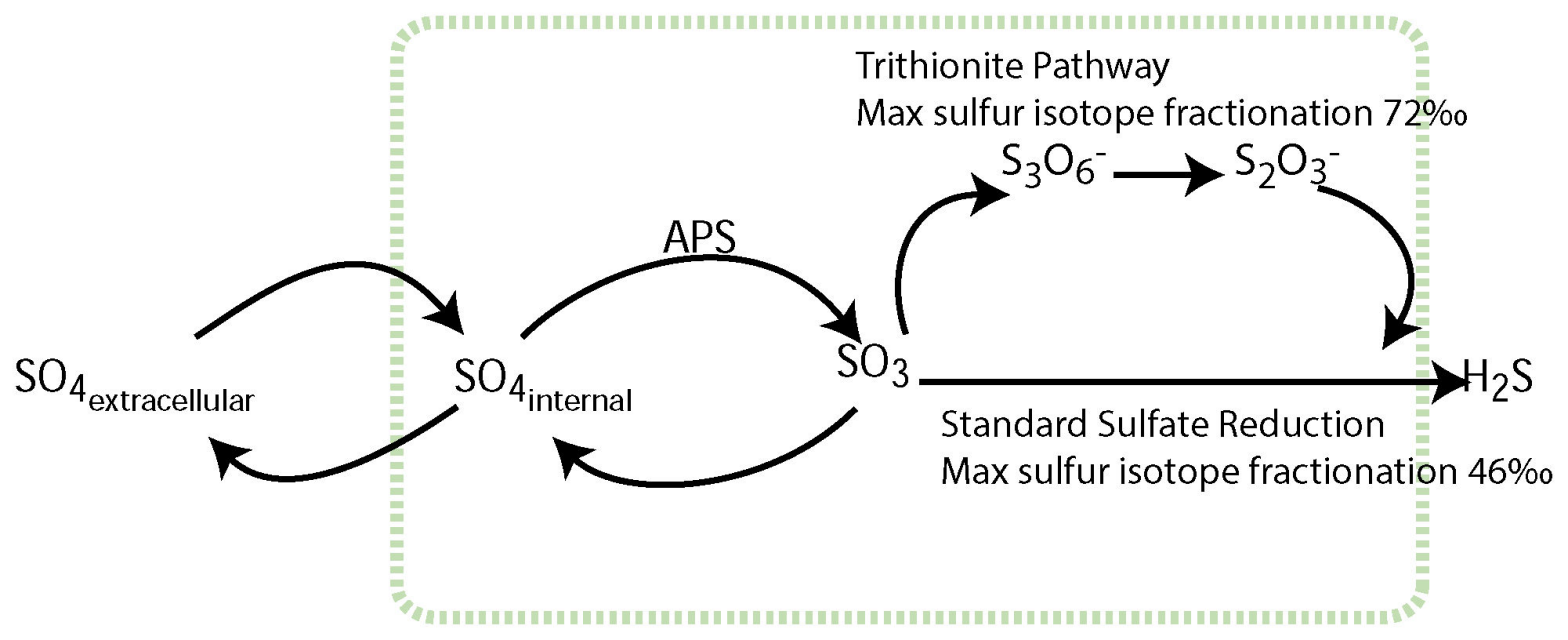
The enzymatic steps involved in bacterial sulfate reduction.

At its simplest level, there are three steps during bacterial sulfate reduction: the incorporation of sulfate into the bacterial cell, the two-step reduction of sulfate to sulfite (SO_3_^2-^) via the APS intermediate, and the reduction of sulfite to sulfide - Figure 1 [33,34]. Sulfur isotopes are negligibly partitioned during the incorporation of sulfate into the cell, and then the two reductive steps (first to sulfite and then to sulfide) can each maximally partition sulfur isotopes up to 25‰. The reduction of sulfite to sulfide can proceed directly or via the trithionite pathway, where the reduction is achieved through three separate enzyme-electron transfers (Figure 1). If sulfate reduction proceeds via this trithionite pathway, the sulfur isotopes can be partitioned even further, reaching a maximum total expressed isotope fractionation of 72‰ [28,35]. Thus the measured difference between the sulfate precursor and the reduced sulfur product (sulfide, elemental sulfur) can yield insight into the biochemical pathway used during sulfate reduction. In this paper we employ this sulfur isotope technique to explore the formation of sulfur nodules in the Lisan Formation near the Dead Sea in Israel. It is these sulfur nodules that form the historical basis for the ‘brimstone’ received from the skies in the biblical story of Sodom and Gomorrah.

## Geological Setting

**Figure 2:**
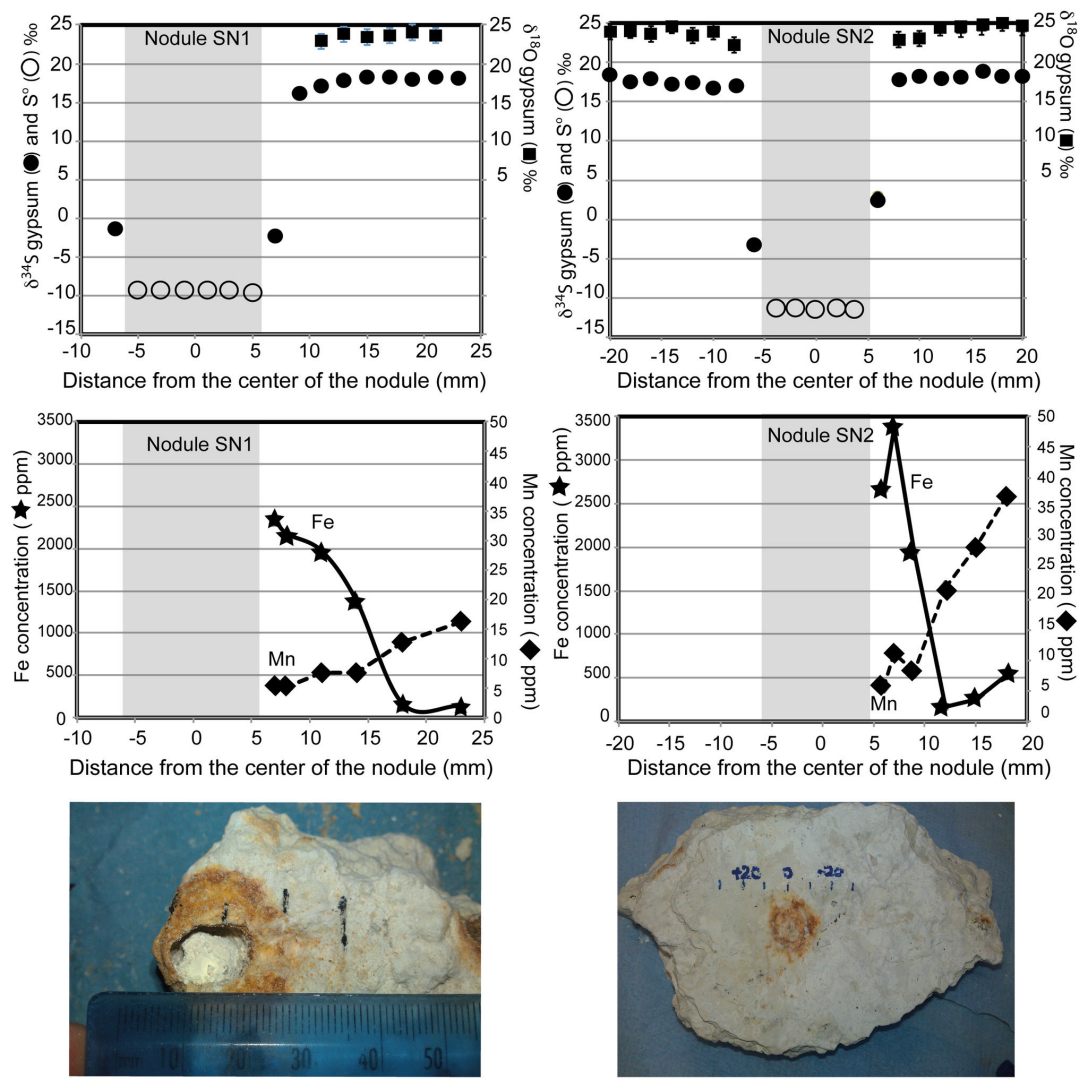
Isotope and major ion results from sulfur nodules 1 (left) and 2 (right). In the top panels we show sulfur isotopes in gypsum (black circles) and elemental sulfur (open circles) and oxygen isotopes in gypsum (black squares). Error bars on the oxygen isotopes in gypsum are based on replicate measurements. In the middle panels we show total iron concentration (stars) and total manganese concentration (diamonds). Photos of the nodules are included at the bottom.

The hypersaline Lake Lisan was the late Pleistocene precursor to the Dead Sea [12]. During its history, 70-14ka BP, it is thought to have alternated between meromictic periods, when the lake was stratified, and holomictic periods, when the lake overturned. During meromictic periods, the less dense upper layer was being replenished by rain and river input [12]. When this stopped, evaporation caused this upper layer to decrease in thickness and become more dense, eventually leading to overturning. This led to gypsum supersaturation, and voluminous gypsum precipitation, which is isotopically homogenous [12,36]. No pyrite has been found in the Lisan Formation [12,36]. It has been suggested that greigite (Fe_3_S_4_) that is currently found in Dead Sea sediments and may also have been present in the Lisan Formation and has since been quantitatively oxidized [37]. The sulfur nodules found in some of the gypsum beds appear to have iron rust rims (Picture, Figure 2).

Our sulfur nodule samples were collected from the Massada M1 section so named in Torfstein et al. (2008) [36]. The study location is at the base of the Massada ancient fortification and culturally important historical Site. The Lisan Formation here is approximately 30 meters thick and consists of three stratigraphic units [36]: a lower member that has a prominent gypsum layer, a middle member which consistes of alternating aragonite, gypsum and silto detritus, and an upper member which contains slightly larger gypsum beds than the middle member. The nodules were found in the prominent gypsum bed in the lower member near the access road to the M1 Section.

## Methods

Two nodules, SN1 and SN2 embedded in gypsum, were analysed for the δ^34^S of the sulfur nodule and gypsum, the δ^18^O of the gypsum sulfate, and the iron and manganese concentrations in the gypsum. The iron and manganese concentrations for the nodule samples and its host rock were measured on a VISTA CCD Simultaneous ICP-AES. Approximately 10ml of deionized water was added to around 2-3 grams of gypsum to rinse water-soluble minerals. 70% nitric acid was then added to the remaining solution to dissolve the remaining solid and obtain total Fe and Mn concentrations. The weighing errors, ±2µg for the samples and ±0.2mg for the solutions, were negligible when added to the VISTA analytical error of 3%.

For the sulfur nodule samples, gypsum was collected manually using fine picking tools. Approximately 3-5 grams of gypsum was dissolved in a 0.5M sodium chloride solution and the sulfate was released into the effluent, where it was precipitated as barite by adding excess barium chloride. The barite was then washed with 6N hydrochloric acid followed by two rinses in deionized water.

The δ^18^O_SO4_ of gypsum was analyzed through pyrolysis of sulfate in a graphite crucible in a Temperature Conversion Element Analyser (TC/EA) at 1450°C and the resulting carbon monoxide joins a continuous helium flow to a Delta Plus mass spectrometer. Isotopic standards (NBS-127, 8.6‰, and a laboratory generated standard EM-B at 16‰) were run before and after the samples to correct for machine drift. For δ^34^S_SO4_ of gypsum and the sulfur nodules the samples were measured via combustion of sulfate to SO_2_ with vanadium pentoxide at 1030°C in a Flash EA coupled to a Delta Plus mass spectrometer. Samples were bracketed by two standards; NBS-127 (20.3‰) and IAEA SO-6 (-34.1‰). The sulfur nodules and gypsum were analysed on a D8 Advance Series 2 X-ray Diffractometer. 1mg samples were crushed to a very fine powder before being mixed with acetone and run on the XRD.

## Results

The δ^34^S measured in the sulfur nodules ranges from -9.0 to -11.2‰ (n=24, Table 1, Figure 2, top left panel). The δ^34^S of the surrounding gypsum is 17.8±0.8‰ in SN1 (n=18) and 17.8±0.6‰ in SN2 (n=32; Figure 2 – note that average measured values are presented in Figure 2 top right panel and all data available in Table 1). There is a possible decrease of ~2‰ in the host gypsum closer to the nodule. The average δ^18^O_SO4_ of gypsum is 23.6±0.4‰ for SN1 and 23.9±0.8‰ for SN2, and the δ^18^O_SO4_ of gypsum decreases towards the nodule by ~1.1–1.5‰ (Table 1, Figure 2). The red/black rims were run more than 10 times on the XRD looking for the presence of pyrite, but only gypsum was found; however pyrite can be very difficult to identify in minute quantities by XRD. The δ^34^S of the gypsum in the rim ranges from -3.50 to 4.29‰ (Table 1, Figure 2, top panels). It is possible that this value reflects contamination from elemental sulfur in the nodule, although all possible precautions were taken to make sure no elemental sulfur was included in the gypsum analyses.

**Table 1:** Sulfur and oxygen isotope data for both sulfur nodules.

**Sample**	**Lithology**	**Distance from nodule centre (mm)**	**δ^18^O_SO4_**	**Replicate Analyses**	**Standard Deviation (1σ)**	**δ^34^S**	**Replicate Analyses**	**Standard deviation (1σ)**
SN1 +23	Gypsum	23				18.1	1	-
SN1 +21	Gypsum	21	23.7	4	0.54	18.3	1	-
SN1 +19	Gypsum	19	24.0	6	0.49	17.9	1	-
SN1 +17	Gypsum	17	23.7	7	0.44	18.3	1	-
SN1 +15	Gypsum	15	23.4	6	0.28	18.3	1	-
SN1 +13	Gypsum	13	23.8	6	0.51	17.9	1	-
SN1 +11	Gypsum	11	22.9	4	0.67	17.1	1	-
SN1 +9	Gypsum	9				16.1	1	-
SN1 +7	Rim	7				-2.3	2	0.66
SN1 +5	Nodule	5				-9.5	1	-
SN1 +3	Nodule	3				-9.3	1	-
SN1 +1	Nodule	1				-9.5	1	-
SN1 -1	Nodule	-1				-9.1	1	-
SN1 -3	Nodule	-3				-9.5	1	-
SN1 -5	Nodule	-5				-9.4	1	-
SN1 -7	Rim	-7				-1.4	2	0.93
SN2 +20	Gypsum	20	24.4	6	0.69	18.2	1	-
SN2 +18	Gypsum	18	25.0	6	0.31	18.2	1	-
SN2 +16	Gypsum	16	24.5	6	0.59	18.9	1	-
SN2 +14	Gypsum	14	24.2	6	0.30	18.1	1	-
SN2 +12	Gypsum	12	24.4	5	0.51	18.1	1	-
SN2 +10	Gypsum	10	23.0	6	0.53	18.2	1	-
SN2 +8	Gypsum	8	22.9	4	0.14	17.8	1	-
SN2 +6	Rim	6				3.1	4	0.93
SN2 +4	Nodule	4				-11.6	1	-
SN2 +2	Nodule	2				-11.2	1	-
SN2 +0	Nodule	0				-11.1	1	-
SN2 -2	Nodule	-2				-11.0	1	-
SN2 -4	Nodule	-4				-11.2	1	-
SN2 -6	Rim	-6				-3.5	1	-
SN2 -8	Gypsum	-8	22.2	6	0.86	16.8	1	-
SN2 -10	Gypsum	-10	23.9	6	0.15	16.7	1	-
SN2 -12	Gypsum	-12	23.4	4	0.31	17.4	1	-
SN2 -14	Gypsum	-14	24.7	4	0.89	17.2	1	-
SN2 -16	Gypsum	-16	23.6	6	0.28	18.0	1	-
SN2 -18	Gypsum	-18	24.1	6	0.36	17.5	1	-
SN2 -20	Gypsum	-20	23.9	4	0.14	18.3	1	-

Total iron concentrations in the gypsum are highest near the nodule and decrease with distance away from the nodule (Table 2, Figure 2, middle panel). The increase in iron concentrations near the nodule for both SN1 and SN2 are relatively linear, reaching ~3400ppm next to the nodule. In contrast, the manganese concentration in the gypsum decreases towards the nodules. This occurs over a greater distance than iron and it is an order of magnitude less abundant, <50ppm. The results for manganese concentration are more variable in SN2 than in SN1 (Table 2, Figure 2 middle panel).

**Table 2:** Iron and Manganese concentration data for both sulfur nodules.

Sample	Distance from centre of nodule (mm)	Concentration of total iron (ppm)	Error in [∑Fe] (ppm)	Concentration of total manganese (ppm)	Error in [∑Mn] (ppm)
SN1 +41	41	280.43	8.41	31.09	0.93
SN1 +38	38	268.42	8.05	35.65	1.07
SN1 +34	34	100.15	3.00	12.82	0.38
SN1 +30	30	145.12	4.35	20.41	0.61
SN1 +26	26	104.88	3.15	13.30	0.40
SN1 +23	23	142.21	4.27	16.14	0.48
SN1 +18	18	162.76	4.88	12.72	0.38
SN1 +14	14	1388.10	41.64	7.53	0.23
SN1 +11	11	1976.40	59.29	7.61	0.23
SN1 +8	8	2179.29	65.38	5.16	0.15
SN1 +7	7	2361.65	70.85	5.48	0.16
SN2 +40	40	194.48	5.83	48.19	1.45
SN2+36	36	63.05	1.89	13.25	0.40
SN2 +32	32	101.91	3.06	12.85	0.39
SN2 +28	28	55.57	1.67	6.97	0.21
SN2 +24	24	160.81	4.82	25.21	0.76
SN2 +21	21	395.86	11.88	22.47	0.67
SN2 +18	18	529.95	15.90	36.97	1.11
SN2 +15	15	259.18	7.78	28.53	0.86
SN2 +12	12	163.54	4.91	22.05	0.66
SN2 +9	9	1942.77	58.28	8.19	0.25
SN2 +7	7	3371.53	101.15	11.13	0.33
SN2 +6	6	2648.39	79.45	5.73	0.17

## Discussion

The δ^34^S of the host gypsum, ~18-19‰, is similar to previously reported values for the Lower Gypsum Unit of the Lisan Formation deposited ~56ka [36]. This constant composition, along with a constant δ^18^O_SO4_ ~23–25‰, supports the theory of the unit’s rapid deposition from a well-mixed, isotopically homogenous lake. The δ^34^S of the sulfur nodules are also similar to previous measurements [36]. It seems therefore that our data are representative of the gypsum and the nodules in the Lisan Formation.

The sulfur isotope difference between the gypsum and the nodule is most certainly related to the partitioning of sulfur isotopes during aqueous phase microbially-mediated sulfate reduction, through which ^32^S is preferentially reduced and thus concentrates in the product. Precipitation of gypsum cannot explain the isotopic variations, since sulfur isotope fractionation between pristine seawater sulfate and the precipitated gypsum is generally less than ±1.5‰ [23]. In this discussion we will first address the possible mechanisms for the formation of sulfur nodules in the Lisan Formation, from which we suggest that our data is most consistent with the termination of bacterial sulfate reduction at S^0^. Then we will discuss biochemically why bacterial sulfate reduction might terminate at S^0^ and the implications for the paleoenvironment during aqueous phase diagenesis.

### Formation of Elemental Sulfur Nodules

There are three primary theories for the microbially mediated formation of sulfur nodules; reduction of sulfate [38], oxidation of sulfide [14,16,18–21] or some form of disproportionation—with the caveat being that these processes must terminate at S^0^ [22,23]. Most previous studies have concluded that the reoxidation of sulfide, terminating at S^0^, is the mechanism behind the formation of sulfur nodules [14,39]. We use our data to conclude instead that elemental sulfur nodules in the Lake Lisan were formed by reduction of sulfate through bacterial sulfate reduction during gypsum diagenesis, terminating at elemental sulfur.

We have several reasons for making this conclusion. One of the primarily reasons is that there is no pyrite found within the formation [12,36]. We would expect, given that we have significant concentrations of iron within the gypsum close to the nodule, if sulfide was formed during bacterial sulfate reduction, then some pyrite should be present. This is because of the high reactivity of sulfide towards iron. It has been suggested that the Lisan Formation sediments, titanomagnetite that was initially laid down is replaced with greigite (Fe_3_S_4_) during anoxic burial diagenesis in the presence of bacterial sulfate reduction and that this greigite could represent the ‘missing sulfide’ that was oxidized to make elemental sulfur [37]. In the currently wet Dead Sea sediments, greigite has been found, although it is completely absent in the Lisan Formation, because this greigite was quantitatively oxidized when the Lisan Formation was exposed to the air [37]. However, greigite itself is not generated from aqueous sulfide; greigite is generated as a precursor to pyrite in settings where bacterial sulfate reduction is terminated prematurely and does not go all the way to sulfide [40]. The presence of griegite in the Lisan Formation does not, therefore, require that sulfide was ever produced during bacterial sulfate reduction. Indeed, the past-presence of greigite in the Lisan formation may be further evidence that bacterial sulfate reduction during microbial diagenesis did not go all the way to sulfide. Alternatively, we would need to invoke the production of sulfide that exists but separated from all local iron; this seems implausible. This leads us to suggest that our data is more consistent with bacterial sulfate reduction terminating at elemental sulfur rather than sulfide oxidation. As discussed in the introduction, this has implications for both the biochemical pathways of sulfate reduction, which impinges on the sulfur isotope composition of the nodules.

**Figure 3:**
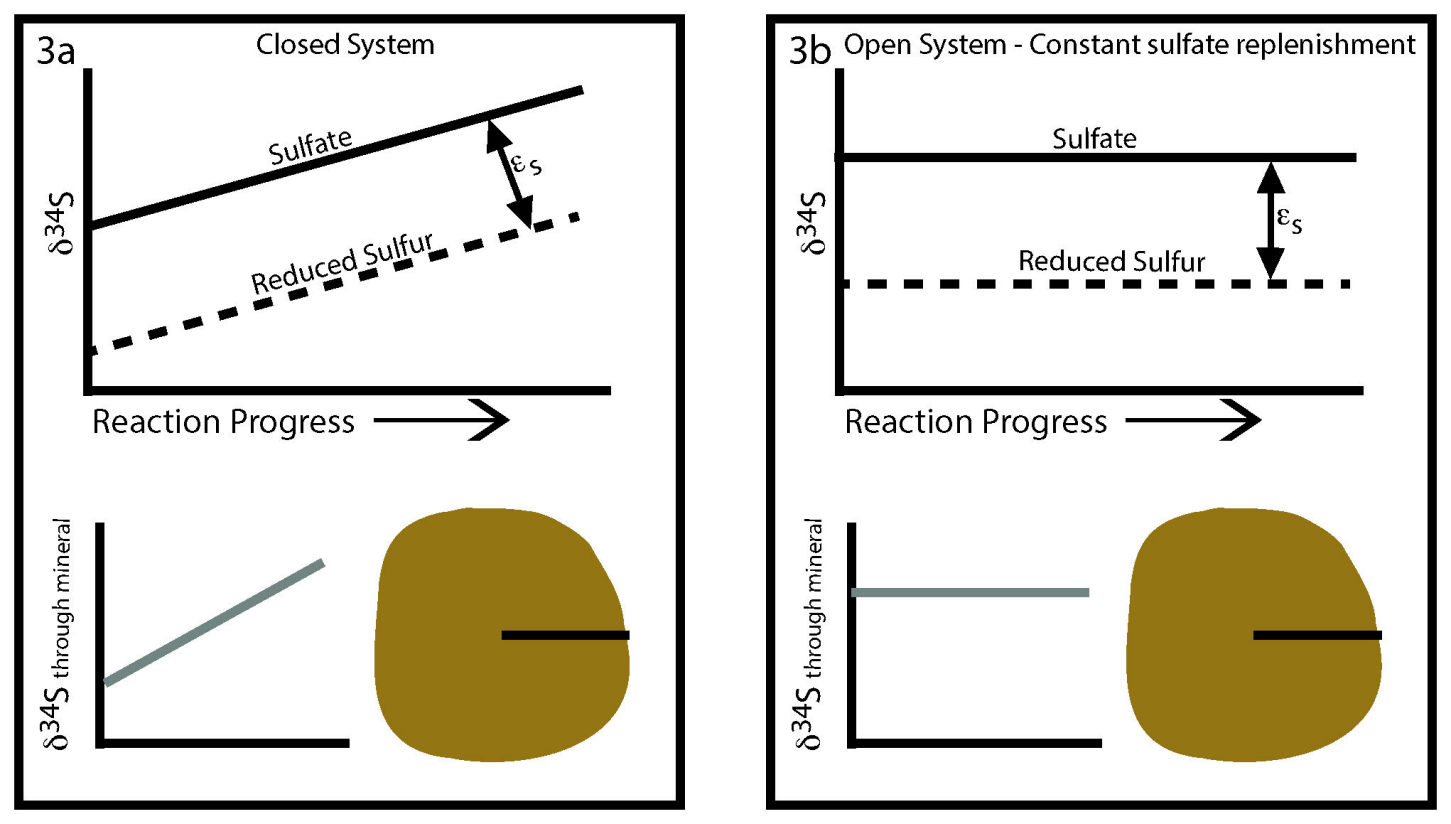
A schematic of the sulfur isotope composition during bacterial sulfate reduction in closed versus open systems. In closed systems the sulfur isotopes in the reduced product (pyrite or elemental sulfur, for example) may have significant isotope variability as they are growing from a pool that is evolving isotopically with time. In contrast in an open system with constant replenishment of the source of sulfate the isotope composition of the reduced product would not be expected to vary. We use our data to conclude that the sulfur nodules in the Lake Lisan formed in an open system. The symbol ε_s_ is the sulfur isotope fractionation during sulfate reduction.

The fact that the sulfur nodules have a uniform isotope composition suggests that they formed in an open system with constant replenishment of sulfate to feed bacterial sulfate reduction. During closed system bacterial sulfate reduction, the continual formation of some reduced sulfur product into some mineral phase results in progressive enrichment in the δ^34^S of the residual sulfate (Figure 3a). This increase in δ^34^S of the residual sulfate in turn can manifest in a progressive δ^34^S across the accreting and growing reduced sulfur mineral phase, typically pyrite. This can result in pyrite crystals that vary by 30-50‰ across a single grain. We can contrast this with open system conditions where the sulfate feeding bacterial sulfate reduction is maintained at a constant δ^34^S because the system is ‘open’ and the aqueous sulfate is continually replenished (Figure 3b). In this case the δ^34^S across the growing crystal is constant and reflects the condition of formation and the sulfur isotope partitioning during the reduction of sulfate and formation of the mineral.

**Figure 4:**
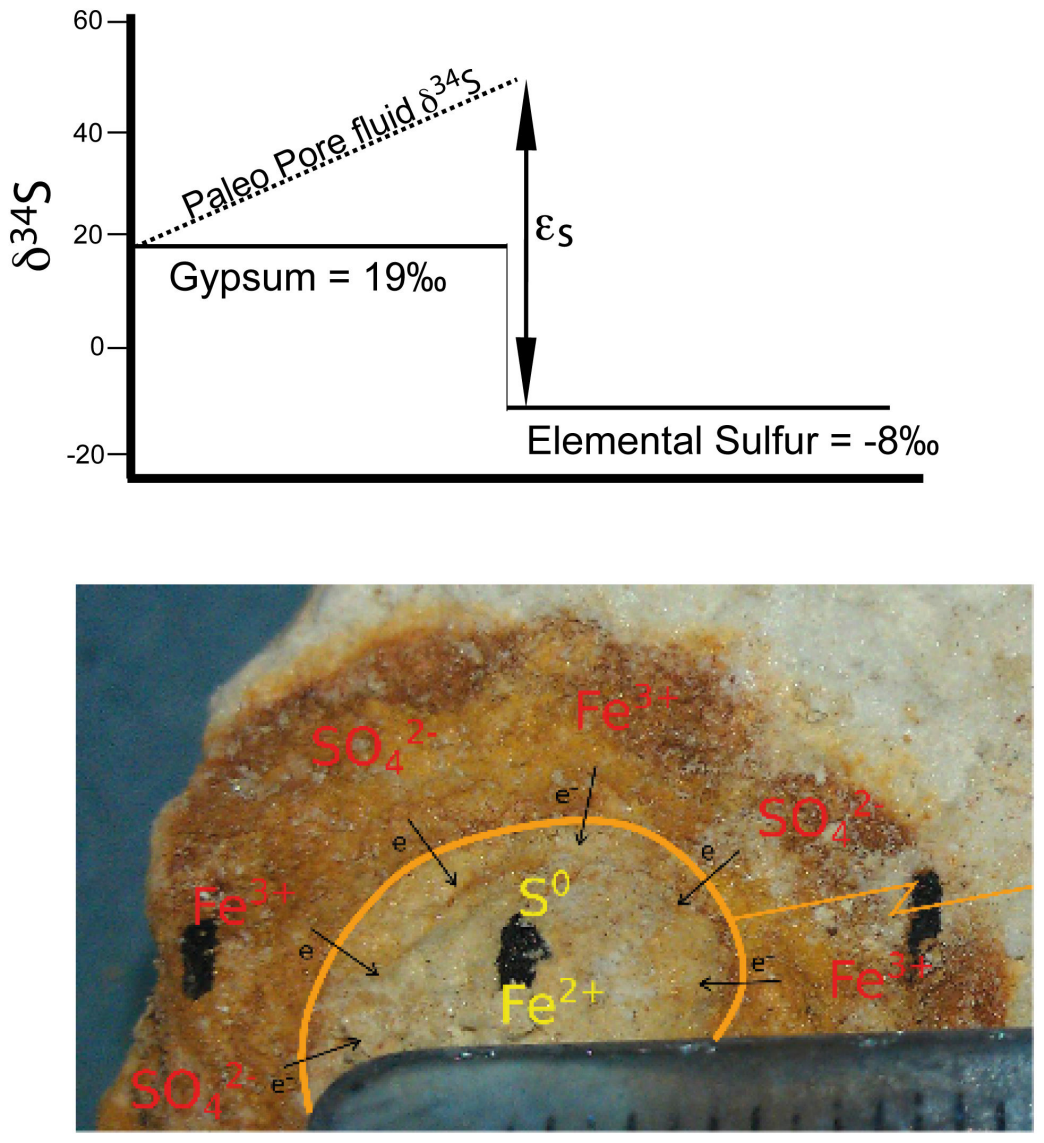
Our hypothesized model for the geochemical environment leading to the formation of the sulfur nodules. Our precursor gypsum is +19‰ and the elemental sulfur is -8‰; these are the two ‘knowns’ as we are able to measure them today. In theory the pore fluid sulfate would be significantly heavier than the gypsum since this would be the mobile pool from which sulfate is reduced to form the sulfur nodule. Thus the sulfur isotope fractionation could be much larger than the 28‰ difference between the gypsum and the nodule.

What is the sulfur isotope fractionation during the formation of the sulfur nodules in the Lisan Formation? While the absolute difference between the gypsum and the sulfur nodules is 27 to 29‰, it is likely that during the formation of the sulfur nodules, the sulfur isotope fractionation was larger than this. Dissolving gypsum in the Lisan Formation during aqueous phase burial diagenesis would have produced initial pore fluid sulfate with a δ^34^S of 18 to 19‰ because there is no initial isotope fractionation during gypsum dissolution. For each mol of this pore fluid sulfate that is reduced, another mol of gypsum would dissolve into the pore fluid to maintain gypsum saturation; this is similar to gypsum beds undergoing aqueous diagenesis today in Guatemalan lakes [14,41]. The pore fluid sulfate would then be the electron acceptor used during bacterial sulfate reduction, and ^32^S (and ^16^O_SO4_) would have been preferentially reduced from this pool. Similar to bacterial sulfate reduction in marine pore fluids, this creates a sulfur isotope gradient in the aqueous sulfate where the sulfate closest to the nodule is isotopically ‘heaviest’ and there is a diffusive gradient to the sulfate further away from the nodule. This suggests that the δ^34^S of the pore fluid sulfate could have been significantly higher than the precursor gypsum; in Lake Peten Itza in Guatemala the pore fluid sulfate δ^34^S is 47 to 50‰ from a precursor gypsum of 18 to 19‰ [14]. A schematic showing this is given in Figure 4.

The nodules’ spherical nature and the appearance of a redox boundary in iron and manganese concentrations near this redox boundary hint that there was an in situ bio-film surrounding an initial nucleation point, although this is not unequivocal. If this was the case, once all the gypsum at the initiation point was consumed, and if it was energetically favorable under the conditions to continue, the bio-film would have expanded, allowing the nodule to gain mass in the centre. The manganese and iron concentration profiles could therefore be read as a snapshot of the redox conditions of the pore fluids at and approaching the nodule-gypsum interface. The increased level of Fe near the nodule hints at the possibility of a dissolved iron pool near the nodule. Part of the Fe(II) could have been oxidized during desiccation, producing the rust colored rims seen in hand specimen (Figure 2). By this token, the low manganese concentrations near the nodule are suggestive of consumption of manganese at the nodule-gypsum interface. We will fit these observations together with a modified model for bacterial sulfate reduction in the next section.

### A modified thiosulfate shunt in unique environments

Our ultimate objective is to use the isotope and major element data to understand the conditions under which bacterial sulfate reduction may result in elemental sulfur as the end product. We suggest that the formation of elemental sulfur in the Lisan Formation results from a modification of the final biochemical step during bacterial sulfate reduction: the six-electron reduction of sulfite to sulfide by dissimilatory sulfite reductase (Dsr). This hypothesis invokes the unique environmental conditions that likely existed in the Lake Lisan sediments during burial-diagenesis: high salinity, supersaturated sulfate in pore fluids, low sulfate reduction rates and low electron potential (not much organic carbon).

**Figure 5:**
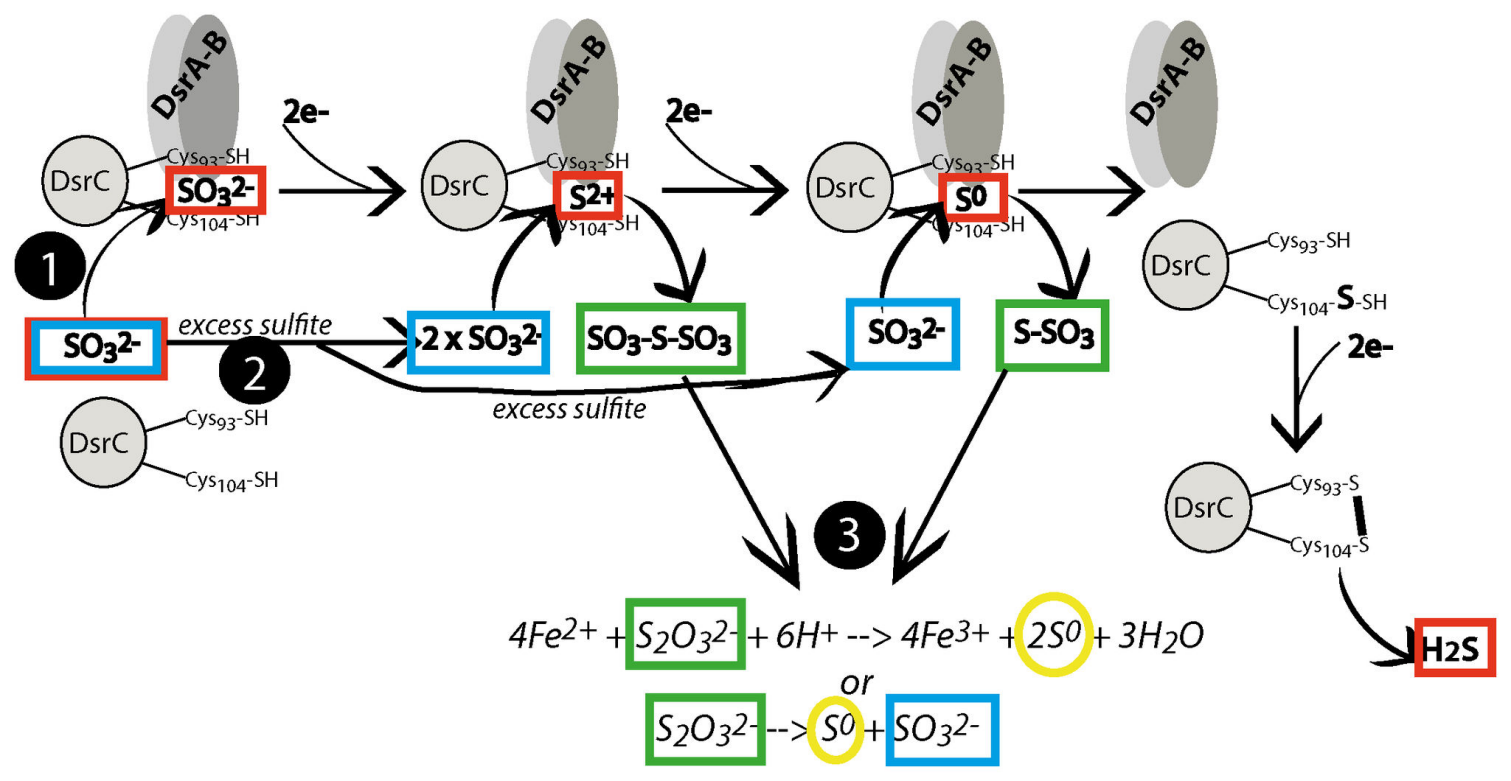
A diagram of the crystal proteins and the proposed pathway to make elemental sulfur. Sulfite is formed during the first two-electron reduction of sulfate within a microbial cell (see Figure 1). Sulfite can be further reduced to sulfide through three two-electron reductions (pathway 1, sulfur species in red boxes). During this, sulfite binds to the DrsA-B and DrsC proteins, where it is sequentially reduced. It has been hypothesized that the DrsC protein plays a particularly important role in the terminal two-electron reduction of elemental sulfur to sulfide. Excess sulfite (blue box) in the cell can further attack S^2+^ and S^0^ while they are bound in the DsrA-B-C complex, forming thiosulfates (green boxes). We propose that these thiosulfates are disproportionated (or oxidized) forming elemental sulfur (yellow circles) and sulfite again, allowing for large amounts of elemental sulfur to accumulate (pathway 3). The specific conditions that permit this to happen are the supersaturated sulfate in the pore fluids coupled with low electron donor.

The recently published crystal structure of dissimilatory sulfite reductase demonstrates that it consists of two proteins, the DsrA and DsrB dimer and DsrC [42] (Figure 5). Sulfite is reduced in the DsrAB-C complex in sequential two-electron transfers; first to S^2+^, then to S^0^ (Figure 5, pathway 1). If either S^2+^ or S^0^ are released from DsrA-B-C, and there is excess intracellular sulfite, then nucleophilic attack by this sulfite on the S^2+^ and S^0^ intermediates will produce S_2_O_6_^2-^ (trithionate) and S_2_O_3_^2-^ (thiosulfate) respectively (Figure 5, pathway 2). Thus intracellular thionate formation is promoted through DsrC inhibition (termination of sulfate reduction before the final two-electron reduction), or excess sulfite supplied to DsrAB relative to the capacity of DsrC to remove the reduced intermediates S^2-^ and S^0^. This is supported by previous work: the accumulation of thionate compounds, particularly thiosulfate, has been observed during bacterial growth both on sulfite and sulfate, as well as when electron supply to DsrC is slow (low electron transfer into the cell by the membrane complex [42-44]. Of the two thionates that can be formed during DsrC inhibition, thiosulfate is much more common [43].

What happens once thiosulfate (or generic thionates) are produced within the cell? Thiosulfate can be subsequently reduced by iron as follows: 4Fe^2+^ + S_2_O_3_^2-^ + 6H^+^ 4Fe^3+^ + 2S^0^ + 3H_2_O. Alternatively, thiosulfate can undergo intracellular disproportionation. A pathway connecting the two species was first suggested by Jørgensen (1990) [45]: S_2_O_3_^2-^ S^0^ + SO_3_^2-^. This latter chemical reaction (disproportionation) also allows recycling of SO_3_^2-^ and therefore repetition of this process to yield higher quantities of S^0^. Although not explicitly written in the former chemical reaction, thiosulfate dispropotionation requires an external electron donor as well, which could be Fe(II) [45]. Thamdrup et al. (1993) [46] suggest a close coupling between thiosulfate and sulfite in marine sediments; thiosulfate concentrations covary with sulfite concentrations when either is found, although neither are particularly abundant in marine sediments. Through the chemical reactions above, the net result of thiosulfate production through DsrC inhibition is the production of elemental sulfur. It might be expected that during bacterial sulfate reduction if excess thionates are produced then elemental sulfur could be an end product.

High production of thionate intermediates during bacterial sulfate reduction has been linked to very low rates of sulfate reduction, likely through the limited supply of electrons to the DsrC dimer preventing the final two-electron reduction [47]. It is possible, therefore, that if our hypothesis for the formation of elemental sulfur nodules is correct, that the rates of sulfate reduction could be very low during the formation of sulfur nodules. Brandt et al. (2001) [7] studied bacteria in the Great Salt Lake, USA, and concluded that very extreme salinities (12% in Great Salt Lake) helped result in low in situ sulfate reduction rates. Recent evidence, however, has suggested that hypersaline conditions in the Dead Sea have not impeded the microbially mediated sulfur cycle (Avrahamov, pers comm). There are many natural environments where sulfate reduction rates are exceptionally low, and sulfur nodules are comparatively rare. Therefore, while low sulfate reduction rates can aid in the formation of sulfur nodules through incomplete reduction to H_2_S via the thionate pathway, it is likely that other factors are also at play in the Lisan Formation.

We hypothesize that the pore fluids that are supersaturated with respect to gypsum may play a role in the modified microbial metabolism and the resulting formation of elemental sulfur. Specifically, a high and sustained supply of aqueous sulfate (it is effectively an infinite reservoir because the pore fluids would have been saturated with gypsum) relative to a small supply of electron donor may poise the system such that intracellular sulfite concentrations are exceptionally high but the terminal reduction cannot occur and therefore the system produces high levels of elemental sulfur. Given the importance of intercellular sulfite on the oxygen isotope evolution of extracellular sulfate [48], this hypothesis could be tested using pore fluid measurements of δ^18^O_SO4_ in modern gypsum bearing sediments where elemental sulfur is forming. Similarly, this intracellular branching point is also key for non-zero Δ^33^S produced during different sulfur related microbial metabolisms [49]. Measurements of Δ^33^S on the nodules could help resolve this speculation of the incomplete termination of bacterial sulfate reduction.

Our invoking the trithionate pathway for bacterial sulfate reduction to form elemental sulfur is consistent with the large sulfur isotope fractionation required by our data (Figure 4). Recent pure culture experiments have shown that at very low sulfate reduction rates and small supply of electrons, sulfur isotope fractionation can often exceed the 46‰ from the straight sulfite reduction classically proposed [28] (Figure 1). Therefore it is possible that at these slow rates of sulfate reduction, the thionates produced from the aborted trithionate pathway could be as much as 50‰ lighter than the precursor sulfite. During the further reduction of thiosulfate either through straight reduction or disproportionation, sulfur isotopes may be partitioned further. Therefore the large isotope difference between gypsum and nodule is consistent with the mechanism we propose. It is however, not inconsistent with the alternative hypothesis that sulfate was reduced all the way to sulfide and then reoxidized to elemental sulfur; the sulfur isotope fractionation during standard bacterial sulfate reduction can, in certain unique environments, be greater than 46‰ and there is very little isotope fractionation on sulfide oxidation back to elemental sulfur.

Given the changes in the iron and manganese concentrations we measure in the gypsum (Figure 2), we favor redox gradients within the aqueous pore fluids surrounding nodule formation that are captured in the trace element data within the gypsum. Our data suggest that at the rim of the nodule, iron is being produced and manganese is being consumed. Iron recycling at the rim itself would generate diffusive gradients of iron within the paleo-pore fluids and would draw iron towards the forming nodule. Specifically we favor the use of iron as an external electron donor coupled to either chemical reaction. Iron oxidation coupled to thiosulfate reduction or disproportionation could be occurring on a bio-film surrounding the area of nodule formation. This reduced iron (Fe(II)) could then be reoxidized through manganese reduction, driving a decrease in the manganese concentrations right next to the nodule. Reduced manganese (Mn(II)) is soluble and would diffuse away from the gypsum-nodule interface, creating the iron/manganese profiles similar to what is measured in modern pore fluids [1]. We measure the total iron and total manganese, therefore redox speciation is difficult to determine. However, the geochemical gradients are, at a minimum, suggestive of redox changes at the nodule-gypsum boundary.

While we prefer the explanation that bacterial sulfate reduction is terminated at elemental sulfur, our data is not wholly inconsistent with full bacterial sulfate reduction followed by partial reoxidation to elemental sulfur. The primary logic against this explanation is that we would need to explain the complete absence of pyrite. We believe that our data showing the very large sulfur isotope fractionation between the gypsum and nodule, coupled with the redox changes near the gypsum-nodule boundary, is more consistent with sulfate reduction terminating at elemental sulfur. It is possible also that some of our data are better explained by a second stage of diagenesis, post-nodule formation. For example, the red staining around the rim could be from the erosion of iron oxide, causing redistribution of some iron oxides to create the ‘halos’ seen (Figures 2 and 4). This could be through simple transportation in pore fluids during desiccation or reduction to water-soluble Fe^2+^ followed by re-oxidation. If the latter is true, the iron (III) reduction could be coupled to the oxidizing of some eroded S^0^.

The slight decrease in the δ^34^S and δ^18^O towards the nodule may also be due to a second phase of diagenesis, post-nodule formation (for example, redistribution of small amounts of elemental sulfur into the gypsum or oxidation of small amounts of elemental sulfur to sulfate during lithification and desiccation). The average δ^18^O of the Lake Lisan was estimated through modelling to be around ~7‰ [50] so if this oxidation proceeded not in the presence of atmospheric oxygen, the resulting sulfate would be close to 7‰ as well (all oxygen atoms come from water). Elemental sulfur oxidation could be coupled to manganese or iron reduction if the redox boundary no longer existed post-nodule formation.

Sulfate reducing bacteria have broad ecological tolerances, and likely were some of the earliest metabolisms on the planet. If our mechanism for elemental sulfur formation is correct, then this is a modern example of how sulfate-reducing bacteria may have their biochemical pathway modified in response to the chemical conditions in unique environments (super saturated sulfate concentrations, low electron donor). There are also testable predictions from this work for sulfur and oxygen isotopes in modern sulfur nodule forming environments, as well as for the less abundant sulfur isotopes, which are sensitive to changes in sulfur flow through the microbial cell.

## Conclusions

In this paper we presented coupled sulfur and oxygen isotope data and major element data from gypsum and sulfur nodules in the Lisan Formation, near the Dead Sea, Israel. The sulfur isotopes were around 27 to 29‰ heavier in the gypsum than in the nodule. The sulfur isotopes in the nodule had all the same value within error, suggesting formation in an open system. We proposed a microbially mediated mechanism for the formation of the sulfur nodules. Specifically, bacterial sulfate reduction typically results in hydrogen sulfide production, however in the Lisan Formation, pyrite has not been found. Therefore, we suggested that in the Lisan Formation, bacterial sulfate reduction terminates instead at elemental sulfur, and that a redox boundary, likely with a biofilm, existed between the gypsum and the nodule which initiated and then gained mass at the center. Elemental sulfur can be produced because of a unique combination of low concentration of organic carbon (low electron donor) and abundant, infinite, aqueous sulfate supply from the gypsum. This, we speculated, creates a larger pool of intercellular sulfite, which attacks the S^2+^ and S^0^ intermediates formed during sulfite reduction, producing thionates. These thionates could be further reduced through iron oxidation or undergo disproportionation to form elemental sulfur. These processes have implications for understaning the adaptations of sulfate reducing bacteria to survive in unique chemical environments.
